# Immunotoxicological Evaluation of* Schinus molle* L. (Anacardiaceae) Essential Oil in Lymphocytes and Macrophages

**DOI:** 10.1155/2018/6541583

**Published:** 2018-10-16

**Authors:** Jonathaline Apollo Duarte, Léa Augusta de Bairros Zambrano, Luciane Dias Quintana, Mariana Balhego Rocha, Elizandra Gomes Schmitt, Aline Augusti Boligon, Marli Matiko Anraku de Campos, Luís Flávio Souza de Oliveira, Michel Mansur Machado

**Affiliations:** ^1^Federal University of PAMPA, Uruguaiana, Rio Grande do Sul, BR 472, Km 585, Caixa Postal 118, Uruguaiana, RS 97500-970, Brazil; ^2^Phytochemical Research Laboratory, Department of Industrial Pharmacy, Federal University of Santa Maria, Build 26, Room 1115, Santa Maria 97105-900, Brazil

## Abstract

*Schinus molle* L. is used to treat various diseases; however, the literature lacks information regarding its possible immunotoxic effects. The aim of the study was to investigate the immunotoxic effects of essential oil from leaves of* Schinus molle L*. in cultures of human lymphocytes and macrophages. The cultures were treated with essential oil (EO) of* Schinus molle* L. and subsequently subjected to genotoxic analysis (comet assay), mutagenic analysis (micronucleus frequency and chromosomal aberration), and cytotoxic (cell viability) and functional parameters (interleukins secretions). Our analyses have determined that the essential oil from leaves of* Schinus molle* L. presents several compounds with *α*-pinene being the major compound; in addition, the compound verbenene was firstly identified; genotoxic effects were detected only in macrophages and only at the two highest concentrations tested. An important finding is that* Schinus molle* L. oil causes an activation of the immune system. This action has its mechanism centered by the cascade nitric oxide-interleukin-10-tumor necrosis factor alpha.

## 1. Introduction

Natural products have been used by humanity since time immemorial [1], and the knowledge of medicinal plants is, in most cases, the only therapeutic resource in many communities and various ethnic groups [2]. Thus, these products can be found as much in the poorest regions of Brazil and in large cities in the country, where they are sold in street markets, popular markets, and are even grown in residential yards [2].

Given this situation,* Schinus molle* L. has been used as a treatment option for the Brazilian population, which relates its use to various activities, such as antiviral and antibacterial usages, treatment of respiratory and urinary infections, and rheumatism alleviation and as an antidepressant [3]. Despite its widespread use in folk medicine, there are no studies in the literature reporting the possible toxic effects of its essential oil (EO).

Reduced resistance to infectious disease was a well-documented consequence of primary and acquired immunodeficiencies, but a novel finding following xenobiotic exposure. The awareness of the consequences of altered immune function is the most likely outcome of inadvertent exposure. The human health implications of studies in which chemical exposure reduced resistance to infection drove an early focus on immunosuppression within the toxicology community [4].

Thus, the importance of this work, which aimed to investigate the possible immunotoxic effects of the plant in question, is evident to corroborate the literature, which lacks such information. To the best of our knowledge, this is the first work with this species to address aspects of cellular, genetic, and functional damage in these cell types.

## 2. Materials and Methods

### 2.1. Botanical Identification of* Schinus molle* L.

The material was attained from a collection of specimen. A voucher specimen was sent to the UFSM Herbarium (Federal University of Santa Maria), identified by the botanical herbarium, and cataloged under the number SMDB-13507.

### 2.2. Essential Oil Extraction of* Schinus molle* L.

The plant material of choice was fresh leaves collected inside the city of Uruguaiana in Rio Grande do Sul state in June 2015. For the extraction, sheets of 30 g were used, which were triturated and subjected to hydrodistillation in a Clevenger apparatus for eight hours. The yield was calculated considering the weight/weight of oil.

### 2.3. Determination of Concentrations

Due to a lack of studies on the plant, doses were chosen to allow a broad-spectrum evaluation, which enabled the determination of a median lethal dose (LD_50_) [5]. Therefore, concentrations of 100 *μ*g/mL, 10 *μ*g/mL, 1 *μ*g/mL, 0.1 *μ*g/mL, and 0.01 *μ*g/mL were initially tested in cultures of lymphocytes and macrophages, and after analysis of cell proliferation, the LD_50_ was determined for the various test cells. The LD_50_ was determined by the statistical method of nonlinear regression for the different cultures grown.

### 2.4. Photochemical Composition

The essential oil was subjected to separation by gas chromatography with detection by mass spectrometry (CG-MS). The analysis was performed on a model 6890N chromatograph coupled with a model 5975B mass detector, both from Agilent Technologies. Chromatographic conditions were as follows: initial oven temperature was 50°C for one minute, followed by heating at 50°C/min to 300°C, keeping this temperature for 9 minutes, for a total of 60 minutes. Separation was achieved on a DB-5MS column (30 m x 320 *μ*m x 0.25 *μ*m) at a constant rate of 1.5 mL/min of helium; detection was performed in quadrupole equipment using ionizing electrons [6]. Identification of components was done based on the retention index (RI), determined by the homologous series of n-alkanes, C7-C30 under similar experimental conditions, compared with the search mass spectra libraries (NIST and WILEY) and mass spectra data in the literature [7].

### 2.5. Lymphocyte Culture

The lymphocyte cultures were prepared using 0.5 ml of venous blood (collected by voluntary venipuncture) and immediately transferred to a culture medium containing 10 ml of RPMI 1640 supplemented with 10% fetal bovine serum (FBS) and 1% streptomycin-penicillin, as described in previous work by our group [5, 8, 9] and approved by the Research Ethics Committee of the Federal University of Pampa (n°. 27045614.0.0000.5323). The cells were placed in a greenhouse at 37°C in a 5% CO2 environment for 72 hours.

### 2.6. Macrophage Culture

The isolation of leukocytes from whole blood was carried out by centrifugation, and the density difference between the leukocyte cells was determined using centrifugation methods and tack plastic/glass monocytes, as described in previous studies [10–12]. Cells of interest were obtained, and the activation of monocytes occurred using lipopolysaccharide (LPS) 1 *μ*g/mL in RPMI 1640 supplemented with 1% penicillin-streptomycin and 25% FBS at 37 ± 1°C and atmosphere containing 5% CO2 and 95% humidity for 2 hours. This exposure resulted in the formation of dendritic cells derived from monocytes to be used in the experiment [13, 14].

### 2.7. Treatments of Cultures

All cultures received the addition of the test compound at a final volume of 100 *µ*L. The groups to be tested were the negative control (NC) phosphate buffer pH 7.4 and the genotoxic positive control with bleomycin 3 *μ*g/ml.* Schinus molle* L. EO at five different concentrations, ranging from their respective LD_50_ to LD_50/10000_, was used for each of the tested cells. For all protocols of the experiment, cultures were analyzed in triplicate (n = 3).

### 2.8. Cytotoxicity of* Schinus molle* L. Oil

The analyzed parameter for evaluation of cytotoxicity was cell viability through the loss of membrane integrity using the trypan blue method [15]. This requires a sample homogenate rate in contact with the trypan blue dye, which stains the viable cells. The analysis was performed using a microscope at 400x. One hundred cells were counted.

### 2.9. Genotoxicity of* Schinus molle* L. Oil

The genotoxicity was evaluated using a comet assay as described by Singh [16]. For this, previously prepared slides covered with high-melting-point agarose with a sample outlet were homogenized with low-melting-point agarose and after being dried were disposed in a vat containing lysing solution for one week. After this process, the slides were subjected to 20 min, 300 mA, 25 V electrophoresis in 300 mM NaOH buffer and pH>13. Held in electrophoresis, the slides were neutralized and dried at room temperature. After drying, the slides were fixed, dried again, rehydrated, stained with silver nitrate solution, and dried at room temperature. The DNA damage was calculated from the cells with different classifications of damage; the damage index ranges from 0 (100 cells x 0, when there was no damage) to 400 (100 cells x 4, when the maximum damage occurred).

### 2.10. Mutagenicity of* Schinus molle* L. Oil

The micronucleus test was the parameter used to evaluate mutagenicity. For this, the cultures were used, and the method described above was performed according to the technique described by Schmid [17] and presented as an index as described by Fenech [18]. Besides this, a chromosomal instability test was also carried out. The chromosomal instability was evaluated by the cytogenetics for Band G, a technique described by Yunis [19], which requires the addition of 10 *μ*L/mL colchicine to each properly treated cell culture. The addition was followed by incubation for 1 hour at 37°C. After incubation, the samples were centrifuged for 10 minutes at 1800 rpm. After centrifugation, the cell pellet was suspended in hypotonic KCl solution and incubated at 37°C for 16 minutes. Another centrifugation was conducted, and the pellet was suspended in acetic acid/methanol (1:3) and again subjected to successive centrifugations. Finally, the pellets obtained were placed onto precleaned slides with 70% ethanol and drying was performed at room temperature. Staining was done with giemsa dye, and the slides were analyzed under a microscope at 1000x magnification.

### 2.11. Interleukins Secretions

For the determination of tumor necrosis factor-*α* (TNF-*α*), interleukine-10 (IL-10), interleukine-6 (IL-6), and nitric oxide production, measurements were made using ELISA kits according to the manufacturer's instructions. All tests were performed in triplicate. The results of these tests were expressed in percentage of production in relation to the negative control.

### 2.12. Statistical Analysis

All analyses were performed using specific statistical software. The analyses were assessed by analysis of variance (ANOVA) followed by* post hoc* Bonferroni test. The results were considered significant at p<0.05.

## 3. Results and Discussion

The yield achieved from EO extraction of* Schinus molle* L. leaves in June/2015 was 0.364%. After determining the yield, work began assessing the phytochemical composition thereof by GC-MS, which resulted in the detection of thirty compounds (Table 1); of these, the highest concentrations found were of *α*-Pinene, Sabinene, Bicyclogermacrene, and Limonene, with percentage of 23.49, 11.36, 10.13, and 9.02, respectively. These four compounds together represent more than 50% of the compounds present and identified in the oil. Other studies have also identified *α*-pinene as a major compound present in the EO of leaves of* Schinus terebinthifolius* R. and* Schinus lentiscifolius* M. [20, 21].

*α*-pinene (2,6,6-trimethylbicyclo [3.1.1] hept-2-ene) is a monoterpene [22] that can be found in different EOs and has various biological uses, such as larvicidals, detergents, and insecticides, among others [23], and numerous studies have sought to elucidate its toxicological profile. Additionally, among the compounds identified, over 65% had already been described in the literature, as compounds present in the leaves of* Schinus molle* L. [3, 20, 24, 25]; however, the  *verbenene* compound was previously identified only in the EO of* Schinus molle* L. fruit and not in its leaves [26], and other compounds, such as citronella, citronella acetate, eugenol, and hexadecane, have not been phytochemically identified for this species before, according to the literature.

**Figure 1:**
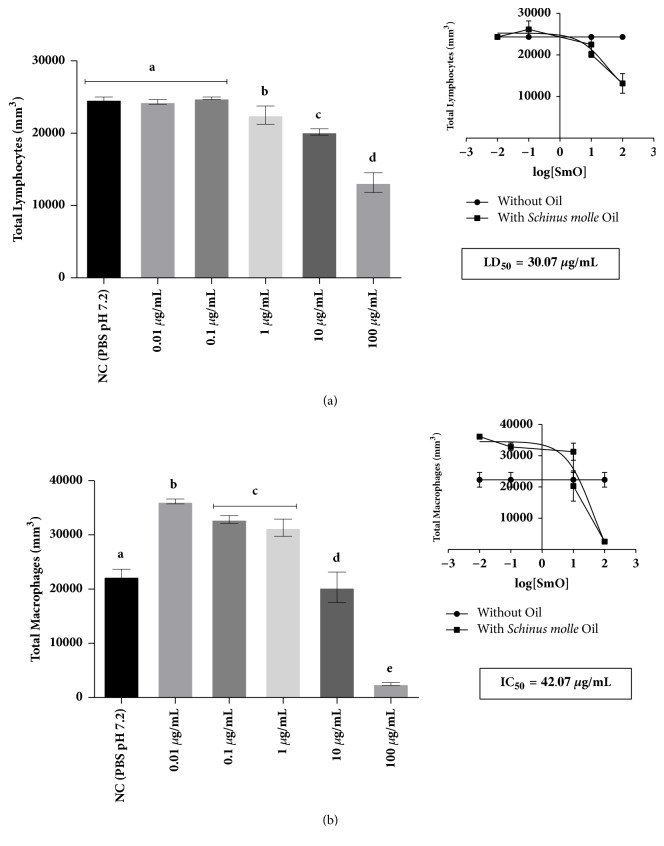
Assessment of cell proliferation for determining the LD_50_ of the essential oil of* Schinus molle* L. culture of (a) human lymphocytes and (b) human macrophages by linear regression. Data expressed as mean ± standard deviation and it was performed at each test triplicate. In each graph the different letters represent a statistically significant difference at p <0.05.

The determination of LD_50_ was carried out per the results found in the analysis of cell proliferation, which was evaluated by the statistical method of nonlinear regression for both types of cell studied. For this, first we made a curve with five EO concentrations in cell cultures, which were 100, 10, 1, 0.1, and 0.01 *μ*g/mL, obtaining at the end of the analysis LD_50_ 30.07 *μ*g/mL for human lymphocytes (Figure 1(a)) and 42.07 *μ*g/mL for human macrophages (Figure 1(b)).

Work performed by Ruffa et al. [27], which investigated the cytotoxic effects of the methanol extract of* Schinus molle* L. in concentrations ranging from 15 to 100 *μ*g/mL for hepatic carcinoma cell cultures, demonstrated a dose-dependent cytotoxic effect and that the LD_50_ of the studied cells was 50 ± 7 *μ*g/mL. Thus, for the development of other tests, the concentrations to be tested were determined, based on LD_50_ found for lymphocytes and macrophages, and these were LD_50_, LD_50/10_, LD_50/100,_ LD_50/1000,_ and LD_50/10000_.

**Figure 2:**
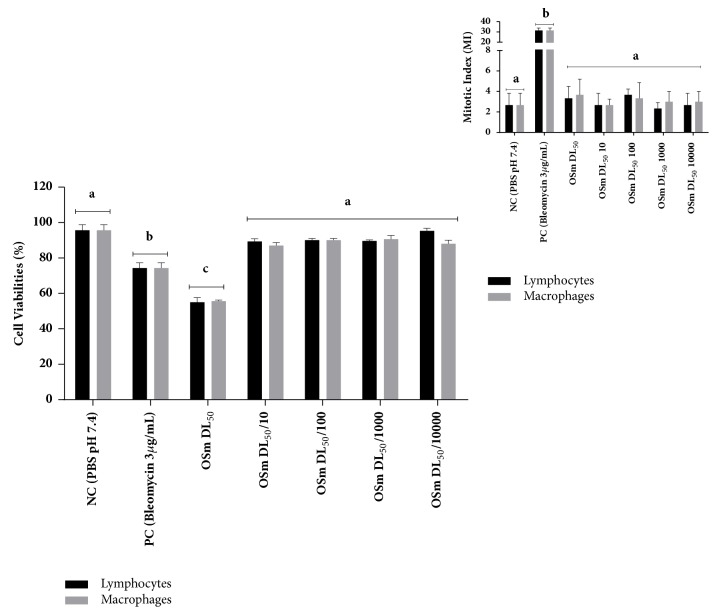
Evaluation of cell viability in cultures of lymphocytes and human macrophages exposed to different concentrations of the essential oil of* Schinus molle* L. Data expressed as mean ± standard deviation and it was performed at each test triplicate (n=3). In each graph the different letters represent a statistically significant difference at p <0.05.

In Figure 2, we can observe the results of cell viability for the cells studied.

The cytotoxicity observed may be related to the presence of certain compounds in the EO, which may promote these cytotoxic effects together and/or in isolation, as described in the study by Sobral-Souza et al. [23], which investigated the cytotoxic effect of the compound *α*-pinene in cultures of fibroblasts at concentrations of 50 and 100 *μ*g/mL. This study found a low cytotoxicity at a concentration of 50 *µ*g/mL in fibroblast cultures. Thus, cytotoxicity could be related to the presence of certain compounds, such as, for example, *α*-pinene, which is the major compound of the EO studied; however, we cannot rule out the possibility that other compounds are contributing to this effect.

Regarding the mitotic index, none of the doses showed effects on the cells tested at these concentrations. Our results for* S. molle *L. are opposed to those found in the study conducted by Pawlowski et al. [20] which investigated the effects of EO extracted from the leaves of* S. terebinthifolius* and* S. molle* in root meristem onion and lettuce and found that the EO metaphase index was reduced. In addition, they showed that the groups treated with* S. terebinthifolius *EO compared to the NC promoted a reduction in the mitotic index of onions and lettuce, higher than 80% and 70%, respectively. The groups treated with* S. molle* L. had a reduced mitotic index for both onions and lettuce, obtaining around 20% and 30%, respectively.

**Figure 3:**
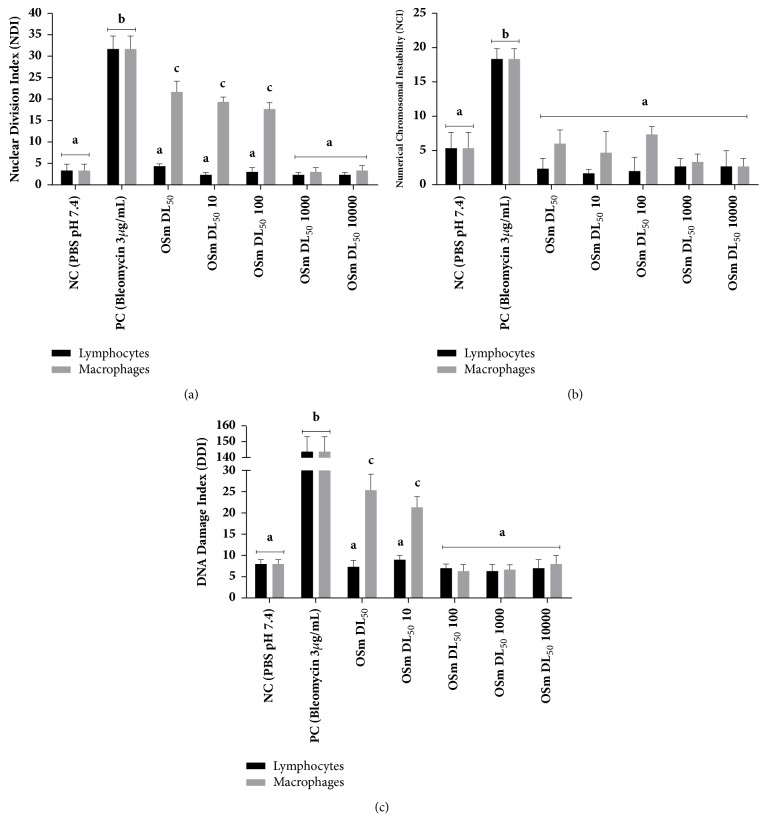
Genotoxic effects of* S. molle* L. essential oil. In (a) the results of the micronuclei (expressed as nuclear division index), in (b) the numerical chromosomal instability, and in (c) the index of DNA damage. Data expressed as mean ± standard deviation and it was performed at each test triplicate (n=3). In each graph the different letters represent a statistically significant difference at p <0.05.

Figure 3 summarizes the genotoxic effects of* S. molle* L. essential oil: in (a) the results of the micronuclei (expressed as nuclear division index), in (b) the numerical chromosomal instability, and in (c) the index of DNA damage.

In the evaluation of DNA damage, only LD_50_ and LD_50/10_ showed significant damage to the macrophages compared to the NC group, representing more than 20% DNA damage each. By contrast, the EO caused no significant DNA damage at any of the concentrations tested in the lymphocytes culture; in the same way it did not change the frequency of micronuclei, certifying that the EO concentrations tested showed no mutagenic effects in the same cells. As for the macrophages, it presented significant, concentration-dependent changes, with a higher frequency of micronuclei compared to the NC for LD_50_, LD_50/10_, and LD_50/100_. These results may be related to the presence of *α*-pinene, which at concentrations of 25, 30, and 35 *μ*M is able to promote significant changes in the frequency of micronuclei and concentration-dependent genotoxicity in hamster cell cultures (V79-Cl3), as described in research by Catanzaro et al. [28]. Türkez and Aydin [29] evaluated the effect of exposure of human lymphocytes to *α*-pinene (10–200 *μ*g/mL) using mutagen parameters (micronucleus test and chromosomal aberration) and concluded that the cells did not change significantly compared to the control. As for the analysis of chromosomal instability developed in this study, the tested concentrations did not promote changes in any of the studied cells, so that they also did not cause changes in the mitotic index for either HLs or HMs compared to the NC.

**Figure 4:**
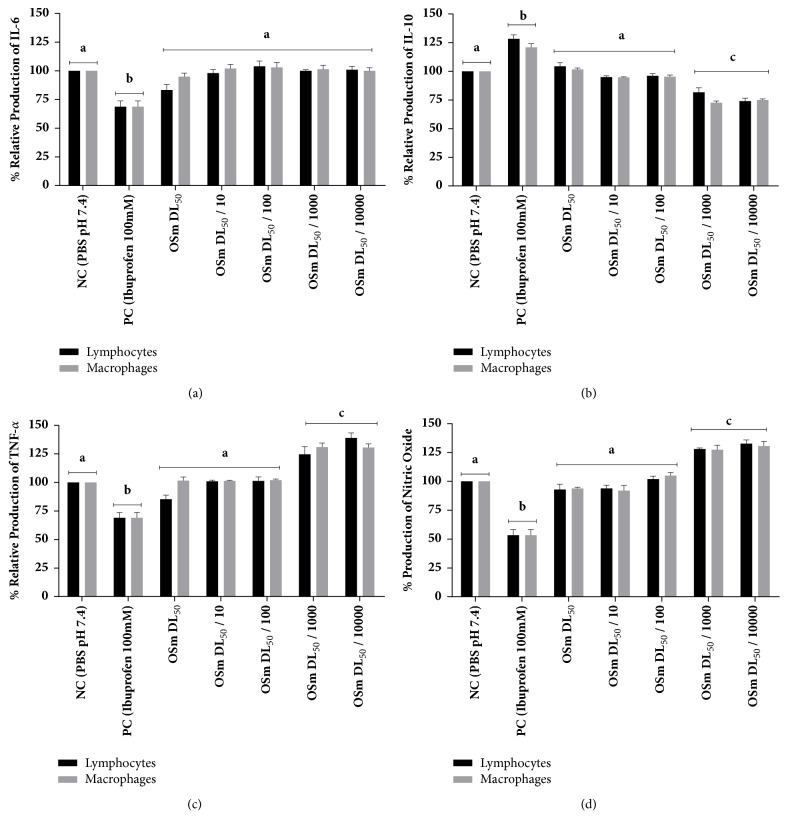
Functional evaluation of the immune system cells. In (a) we have the results of interleukin-6 secretion, in (b) the secretion of interleukin-10, in (c) the production of tumor necrosis factor alpha, and in (d) the production of nitric oxide (NO). Data expressed as mean ± standard deviation and it was performed at each test triplicate (n=3). In each graph the different letters represent a statistically significant difference at p <0.05.

Figure 4 shows functional evaluation parameters of the immune system cells. In (a) we have the results of interleukin-6 secretion, in (b) the secretion of interleukin-10, in (c) the production of tumor necrosis factor alpha, and in (d) the production of nitric oxide (NO).

Cytokines are proteins, which are engaged in the communication between cells of the immune system. IL-6 is a 184-amino acid glycosylated protein, which can be synthesized and secreted by many cell types including monocytes, T-cells, fibroblasts, and endothelial cells. IL-6 binds to a specific receptor (IL-6R), an 80 kDa. During inflammation or infection, IL-6 is secreted by neutrophils, monocytes, macrophages, fibroblasts, endothelial cells, smooth muscle cells, and T-cells type I transmembrane protein [30]. In our study, none of the concentrations tested caused alterations in the relative production of IL-6, both in lymphocytes and in macrophages. Interleukin-10 (IL-10) is a potent anti-inflammatory cytokine that was originally labeled cytokine synthesis inhibitory factor due to its ability to inhibit production of TNF-*α*. These properties prompted early and extensive efforts to utilize IL-10 to modulate immune response in humans. Almost all leukocytes, including T and B cells, dendritic cells, NK cells, mast cells, neutrophils, eosinophils, and keratinocytes produce IL-10 [31]. Tumor necrosis factor (TNF-*α*) is a central proinflammatory cytokine involved in various inflammatory conditions. TNF is a trimeric protein, expressed from a wide variety of cells, and exists in both soluble and membrane-associated forms [32]. Our results showed that there was a reduction in IL-10 production against low oil concentrations and this is reflected in an increase in the production of TNF-*α*, which are directly related. Nitric oxide (NO) is a molecule utilized throughout the animal kingdom as a signaling or toxic agent between cells. Generated by many cell types in a variety of tissues, in mammals it acts as a vascular relaxing agent, a neurotransmitter, and an inhibitor of platelet aggregation. NO plays several roles in immunity—as a toxic agent towards infectious organisms, an inducer or suppressor of apoptosis, or an immunoregulator [33]. Few works report the relationship between the* Schinus* genus and immune system mediators.

Our work is the first to evaluate these markers in these cells. We can infer that the results obtained are a cascade of effects. Oil components such as *α*-pinene, which are known to have this action (REF), inflame the production of nitric oxide, which in turn inhibits the production of interleukin-10 and results in increased production of tumor necrosis factor alpha. By adding these actions, we have an immune response activation by the components of the oil. Our study needs to determine whether this immune activation would be beneficial (in the case of a foreign agent) or harmful to the body (in a mechanism like an autoimmune disease) [34–36].

## 4. Conclusion

The essential oil from leaves of* Schinus molle* L. presents various compounds, *α*-pinene being the major compound; in addition, the compound verbenene was first identified in the essential oil of the leaves of* Schinus molle *L. The EO promoted cytotoxicity in both types of cell tested, but genotoxic effects were detected only in macrophages and only at the two highest concentrations tested; as compared to the mutagenic effects, the EO did not cause chromosomal alterations or alterations in the index of cell division but did lead to an increase in the frequency of concentration-dependent micronuclei of macrophages. An important finding is that* Schinus molle *L. oil causes an activation of the immune system. This action has its mechanism centered by the cascade nitric oxide-interleukin-10-tumor necrosis factor alpha. However, it is worth noting the importance of knowledge of the EO constituents of* Schinus molle *L. to obtain further clarification concerning its toxicity, as this plant is widely used in folk medicine to treat various infirmities.

## Figures and Tables

**Table 1:** Composition of essential oil of *S. molle *L.

Compounds	RI^a^	RI^b^	Percentage
*α*-Pinene	941	939	23.49 ± 0.2
Sabinene	978	976	11.36 ± 0.14
Bicyclogermacrene	1495	1494	10.13 ± 0.08
Limonene	1030	1031	9.02 ± 0.09
Spathulenol	1576	1576	6.59 ± 0.17
*β*-pinene	980	980	6.09 ± 0.54
*β*-caryophyllene	1417	1418	5.28 ± 0.36
Germacrene-D	1480	1480	4.21 ± 0.47
Eugenol	1356	1356	2.96 ± 0.08
Myrcene	991	991	2.83 ± 0.09
*α*-Bisabolene	1504	1504	2.57 ± 0.07
1.8 cineol	1035	1033	2.04 ± 0.02
Terpinen-4-ol	1177	1177	1.74 ± 0.03
*α*-Humulene	1451	1454	1.73 ± 0.01
*α*-Terpinene	1019	1018	1.46 ± 0.06
Hexadecane	1600	1601	1.07 ± 0.9
Citronellal	1153	1153	1.05 ± 0.08
Germacrene-B	1557	1556	1.02 ± 0.01
Citronellyl acetate	1354	1354	0.83 ± 0.02
Verbenene	969	967	0.74 ± 0.3
Aromadendrene	1442	1439	0.65 ± 0.2
*δ*-terpinene	1063	1062	0.38 ± 0.1
*α*-Cadinene	1538	1538	0.35 ± 0.06
Camphene	953	953	0.27 ± 0.08
p-Cymene	1023	1026	0.25 ± 0.09
Caryophyllene oxide	1580	1581	0.25 ± 0.05
(E)-*β*-cymene	1052	1050	0.16 ± 0.01
*γ*-Cadinene	1523	1521	0.09 ± 0.02
*α*-Thujene	930	931	0.08 ± 0.01
*α*-Terpineol	1191	1189	0.07 ± 0.03

Total identified (%)			98.76 ± 0.08

Relative proportions of the essential oil constituents were expressed as percentages ± SD (n=3).

^a^Experimental retention indices (based on homologous series of n-alkane C_7_-C_30_).

^b^Retention indices from the literature (Adams, 1995).

## Data Availability

Any and all data files are available for evaluation as per request to the corresponding author's e-mail.
